# Priming approaches to improve the efficacy of mesenchymal stromal cell-based therapies

**DOI:** 10.1186/s13287-019-1224-y

**Published:** 2019-05-02

**Authors:** Nádia de Cássia Noronha NC, Amanda Mizukami, Carolina Caliári-Oliveira, Juçara Gastaldi Cominal, José Lucas M. Rocha, Dimas Tadeu Covas, Kamilla Swiech, Kelen C. R. Malmegrim

**Affiliations:** 1 Center for Cell-based Therapy, Regional Blood Center of Ribeirão Preto, Ribeirão Preto Medical School, University of São Paulo, Ribeirão Preto, Brazil; 2 Graduate Program on Bioscience and Biotechnology, School of Pharmaceutical Sciences of Ribeirão Preto, University of São Paulo, Ribeirão Preto, Brazil; 3 In Situ Cell Therapy, SUPERA Innovation and Technology Park, Ribeirão Preto, São Paulo Brazil; 4 Graduate Program on Basic and Applied Immunology, Ribeirão Preto Medical School, University of São Paulo, Ribeirão Preto, Brazil; 5 Department of Pharmaceutical Sciences, School of Pharmaceutical Sciences of Ribeirão Preto, University of São Paulo, Ribeirão Preto, Brazil; 6 Department of Clinical, Toxicological and Bromatological Analysis, School of Pharmaceutical Sciences of Ribeirão Preto, University of São Paulo, Avenida do Café, s/n°, Ribeirão Preto, SP 14010-903 Brazil

**Keywords:** Mesenchymal stromal cells, Priming, Cell therapy, Pro-inflammatory cytokines, Pharmaceutical drugs, Biomaterials, Culture conditions

## Abstract

Multipotent mesenchymal stromal cells (MSC) have been widely explored for cell-based therapy of immune-mediated, inflammatory, and degenerative diseases, due to their immunosuppressive, immunomodulatory, and regenerative potentials. Preclinical studies and clinical trials have demonstrated promising therapeutic results although these have been somewhat limited. Aspects such as low in vivo MSC survival in inhospitable disease microenvironments, requirements for ex vivo cell overexpansion prior to infusions, intrinsic differences between MSC and different sources and donors, variability of culturing protocols, and potency assays to evaluate MSC products have been described as limitations in the field. In recent years, priming approaches to empower MSC have been investigated, thereby generating cellular products with improved potential for different clinical applications. Herein, we review the current priming approaches that aim to increase MSC therapeutic efficacy. Priming with cytokines and growth factors, hypoxia, pharmacological drugs, biomaterials, and different culture conditions, as well as other diverse molecules, are revised from current and future perspectives.

## Background

Multipotent mesenchymal stromal cells (MSC) are components of the tissue stroma of all adult organs and, in all probability, are located at perivascular sites, where they play an important role in tissue homeostasis, surveillance, repair, and remodeling [1–3]. MSC are a heterogeneous cell population characterized by spontaneous adherence to plastic; they have a typical immunophenotypic profile (expression of surface markers CD44, CD73, CD90, and CD105, and lack of CD34, CD45, CD14, and HLA-DR) and multilineage-differentiation potential into osteocytes, adipocytes, and chondrocytes [4]. MSC isolated from different tissue sources show different cellular composition, lineage-specific differentiation potential, and self-renewal capabilities [4].

MSC have been investigated in cell-based therapies because of their remarkable anti-inflammatory, immunosuppressive, immunomodulatory, and regenerative properties [5, 6], which involve both paracrine and cell-to-cell contact mechanisms. Paracrine effects depend on MSC secretome, which includes many bioactive molecules, such as growth factors, cytokines, chemokines, and microvesicles/exosomes carrying proteins and/or miRNAs to target cells [5–7].

MSC secretome also contains large amounts of immunoregulatory factors, which are capable of modulating innate and adaptive immune responses [6]. One of the major immunosuppression mechanisms of human MSC is the production of indoleamine-2,3-dioxygenase (IDO). IDO is involved in the l-tryptophan catabolism leading to its depletion in the surrounding microenvironment and accumulation of kinurenin, which then inhibits T cell activation, proliferation, and functional activity of T cells, DCs, and NK cells and Th17 differentiation, among other effects [8]. Several molecules produced by MSC are able to influence (suppress or modulate) the immune responses, such as transforming growth factor (TGF)-β1, hepatocyte growth factor (HGF), prostaglandin-E2 (PGE2), interleukin-6 (IL-6), interleukin (IL-10), nitric oxide (NO), human leukocyte antigen-G molecules (HLA-G5), and leukemia inhibitory factor (LIF). The detailed mechanisms by which these molecules suppress or modulate the immune cells are still not completely understood and are beyond the scope of the review. Another important mechanism thereby MSC suppress and/or modulate the immune response is via generation and/or expansion of immune regulatory cells [6].

Under homeostatic conditions, MSC express low levels of class I major histocompatibility complex (MHC) molecules and do not express class II MHC and costimulatory molecules (CD40, CD80, and CD86). Therefore, in homeostatic conditions, MSC are considered hypoimmunogenic and have immune evasion abilities, which make them suitable for allogeneic transplantation settings [8]. However, under inflammatory conditions, mainly enriched by pro-inflammatory cytokines as interferon-γ (IFN-γ), tumor necrosis factor-α (TNF-α), interleukin-17 (IL-17), and interleukin-1 (IL-1β), MSC are “licensed/activated/primed,” thereby upregulate class I/II MHC and costimulatory molecules, display improved proliferation and survival conditions, and acquire enhanced immunomodulatory and immunosuppressive functions, as further detailed reviewed in the next section.

MSC are capable of migrating to inflammatory sites due to high expression of chemokine receptors, matrix metalloproteinases (MMPs), and adhesion molecules [4]. Their important role in tissue regeneration is based on (i) the “empowering” of resident cells (such as fibroblasts, endothelial cells, and tissue progenitors) by the secretion of bioactive molecules that induce cellular proliferation and differentiation and (ii) the differentiation into functional mesodermal cells which replace damaged cells [5, 9].

In summary, the therapeutic potential of MSC is attributed to their ability to undergo lineage-specific differentiation, to modulate the immune system, and to secrete important bioactive factors [10]. Therefore, MSC are very attractive candidates for cell-based therapies in immune-mediated, inflammatory, and degenerative diseases [5, 9]. Indeed, a huge number of preclinical studies and about 900 clinical trials have been reported in the past 10 years (source: http://www.clinicaltrials.gov); however, many of them have shown therapeutic failure, especially in humans [6, ﻿7﻿].

MSC properties are influenced by in vivo and in vitro biological, biochemical, and biophysical factors, which tightly regulate MSC functions and survival [11] through reciprocal interactions between the cells, extracellular matrix (ECM), and soluble bioactive factors. MSC interact with surrounding tissues and cells in a three-dimensional space, regulating the ECM, therefore promoting angiogenesis, producing anti-inflammatory molecules, preventing cell death (anti-apoptotic effects), and modulating the immune system [12]. In this context, the major current challenge in MSC-based therapy is to develop in vitro culture methods that mimic the natural MSC niche, while at the same time allowing cell expansion at a clinical-grade scale, not compromising cell quality attributes and function.

To date, several studies have demonstrated that the modulation of biological, biochemical, and/or biophysical factors can influence MSC fate, lineage-specific differentiation, and functions and also enhance their therapeutic potential [12, 13]. One of the first reported approaches was *cell priming* (also referred to as licensing or preconditioning) with pro-inflammatory mediators [11, 14–17]. Cell priming consists of preparing cells for some specific function or lineage-specific differentiation, which involves cell activation, molecular signaling, genetic or epigenetic modifications, and morphology/phenotype changes. This concept is commonly used in the immunology field, and it has been adapted for the stem cell scope. For example, pro-inflammatory cytokine (such as interferon-γ) may be added to the medium during MSC culture to augment their anti-inflammatory effects [16].

**Fig. 1:**
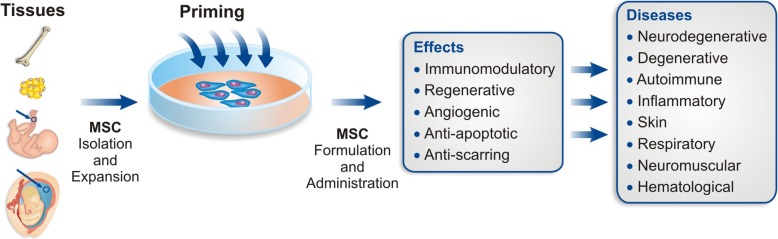
Overview of the production of primed MSC for the treatment of different disease types. Six steps for primed MSC production are indicated: tissue source selection, MSC isolation, MSC priming (the four main classes of priming approaches currently available are represented), MSC expansion, MSC product formulation, MSC administration, and application in different disease types. The rationale is to use different MSC sources/priming approaches for different clinical applications

Several priming approaches have been proposed in the last years to improve MSC function, survival, and therapeutic efficacy [14]. Here, we have divided these approaches into five categories: (a) MSC priming with inflammatory cytokines or mediators, (b) MSC priming with hypoxia, (c) MSC priming with pharmacological drugs and chemical agents, (d) MSC priming with biomaterials and different culture conditions, and (e) MSC priming with other molecules (Fig. 1). In this comprehensive and updated review, we address available priming approaches and discuss their potentials and limitations, as well as the perspectives of this research field.

## MSC priming with cytokines

**Fig. 2:**
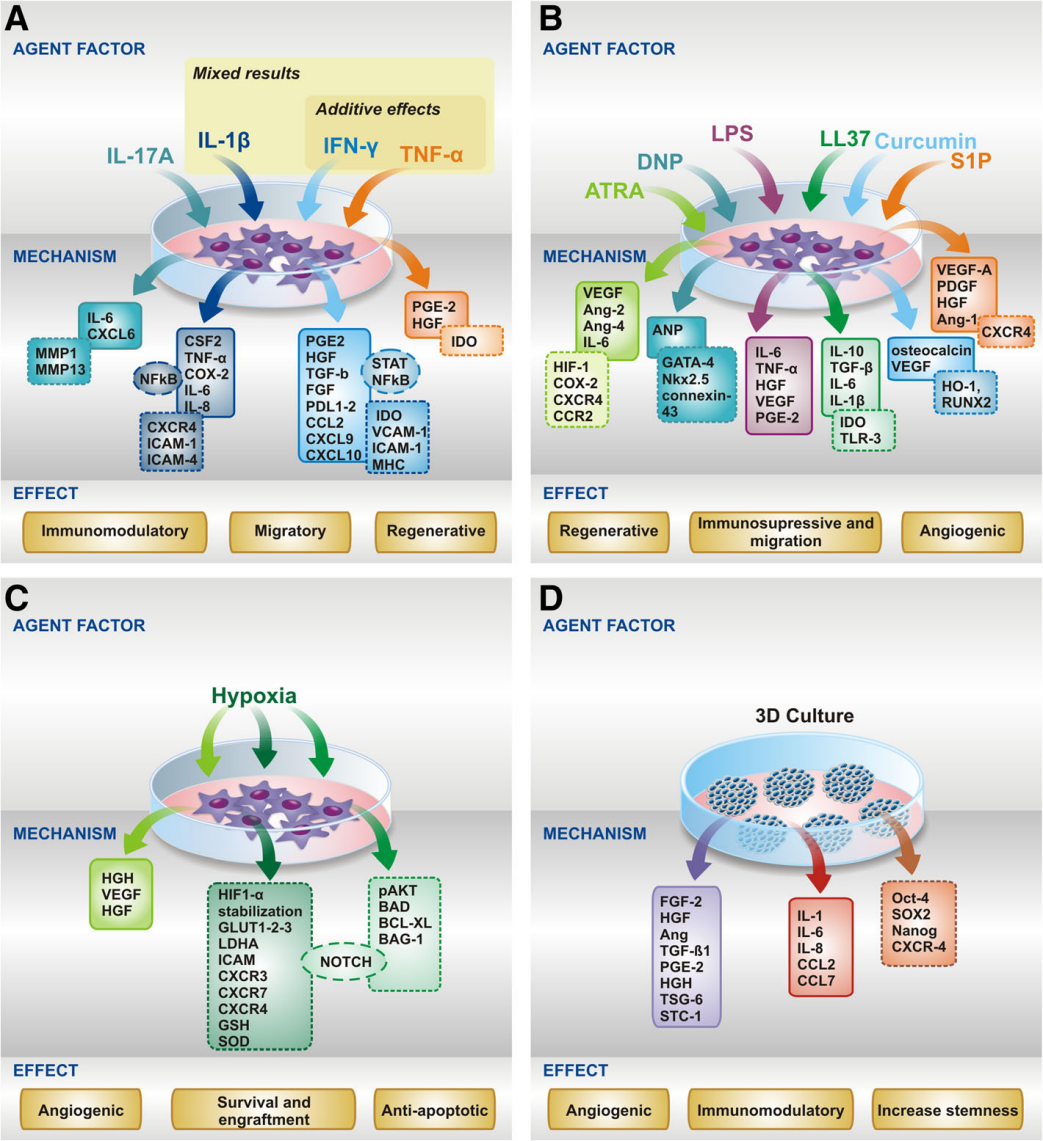
Schematic representation of the main priming approaches to improve MSC therapeutic efficacy**.** Priming with **a** cytokines or growth factors, **b** pharmacological or chemical agents, **c** hypoxia, **d** 3D culture conditions. Priming factors/agents and their respectively triggered mechanisms are linked by arrows and boxes of the same color. Released soluble factors are represented in continuous-line boxes, while other upregulated molecules (such as transcription factors, metalloproteinases, chemokine receptors, and enzymes) are represented in dashed-line boxes. The general priming effects on MSC (immunomodulatory, migratory, regenerative, immunosuppressive and migration, angiogenic, survival and engraftment, anti-apoptotic, increase stemness) triggered by the priming factor/agent are indicated in yellow boxes at the bottom of each figure

Many studies have demonstrated the effects of MSC priming with pro-inflammatory cytokines or growth factors. This strategy aims to improve the immunosuppressive function and to increase their secretion of anti-inflammatory and immunomodulatory factors [11, 14–16] (Table 1, Fig. 2).

**Table 1:** Priming of MSC with cytokines and growth factors

Stimuli	Source MSC	Model/disease	In vivo*/*in vitro	Results	References
IFN-γ and TNF-α	Bone marrow	–	In vitro	Induced chromatin remodeling in the IDO1 promoter.	[33]
IFN-γ and TNF- α	Bone marrow	–	In vitro	Suppressed T cell proliferation by IDO upregulation and induced greater IL-10-secreting M2 macrophages differentiation.	[31]
IFN-γ and TNF- α	–	–	In vitro	Increased factor H production.	[30]
IFN-γ	Bone marrow	–	In vitro	Inhibited T cell effector function through the ligands for PD1 and Th1 cytokines production.	[20]
IFN-γ	Bone marrow	–	In vitro	Retained the ability to inhibit the degranulation and proliferation of cytotoxic T cells post-thaw.	[24]
IFN-γ	Bone marrow	–	In vitro	Reestablished immunosuppressive effect on T-cell proliferation and did not upregulate HLA-DR of senescent MSC.	[25]
IFN-γ	Bone marrow	DSS-induced colitis model	In vitro*/*in vivo (mice)	Attenuated development of colitis, reduced pro-inflammatory cytokine levels in colon and increased migration potential.	[26]
IFN-γ	Umbilical cord	Healthy donor/tissue	In vitro	Increased suppression of NK cells and reduced NK-mediated cytotoxicity.	[21]
IL-1α and IL-1β	Bone marrow	–	In vitro	Increased secretion of G-CSF through IL-1 receptor type 1, reduced the secretion of IL-6 and TNF-α in microglial cells.	[53]
IL-1β	Umbilical cord	DSS-induced colitis model	In vitro*/*in vivo (mice)	Attenuated the development of murine colitis, increased migration potential to inflammatory sites by CXCR4 upregulation.	[52]
IL-1β	Bone marrow	Healthy donor/tissue	In vitro	Induced the secretion of trophic factors and adhesion to ECM components; enhanced recruitment of leucocytes by NF-κB pathway.	[51]
FGF-2	Dental pulp	Subcutaneous implantation of priming MSCs encapsulated in a 3D collagen matrix	In vitro*/*in vivo (mice)	Increased VEGF and HGF secretion and improved vascularization in vivo*.*	[56]
TNF-α and LPS	Bone marrow	–	In vitro	Increased alkaline phosphate activity and bone mineralization.	[50]
IL-17A	Bone marrow	–	In vitro	Increased suppressive potential of T cell proliferation correlated with increased IL-6, inhibited surface CD25 and Th1 cytokines expression, and induced iTregs.	[164]

*IFN-γ* interferon-gamma, *TNF-α* tumor necrosis factor-alpha, *IL-1β* interleukin-1 beta, *FGF-2* fibroblast growth factor-2, *IL-1α* interleukin-1 alpha, *LPS* lipopolysaccharide, *IL-17A* interleukin-17A

### IFN-γ priming

Priming or preconditioning with IFN-γ enhances the immunosuppressive properties of MSC. Upon IFN-γ priming, MSC upregulate IDO, secrete important immunomodulatory molecules, such as PGE2, HGF, TGF-β, and CCL2, increase the expression of class I and class II histocompatibility leucocyte antigen (HLA) molecules and of co-stimulatory molecules [18].

Preconditioning of Warton’s jelly-derived MSC (WJ-MSC) with IFN-γ leads to the upregulation of immunosuppressive factors (IDO and HLA-G5), chemokine ligands (CXCL9, CXCL10, and CXCL11), and adhesion proteins (VCAM-1 and ICAM-1). It has been demonstrated that upon co-culturing of IFN-γ-primed MSC with activated lymphocytes, there is decreased production of IFN-γ and TNF-α, increased secretion of interleukin-6 (IL-6) and interleukin-10 (IL-10), increased frequency of CD4^+^CD25^+^CD127^dim/−^ T cells, and decreased frequency of Th17 cells [19].

MSC primed with IFN-γ also inhibit T-cell effector functions through the upregulation of programmed cell death-1 ligands (PDL-1), at the same time, but independently of IDO upregulation [20].

Noone and coworkers demonstrated that IFN-γ-preconditioned MSC suppressed NK activation more efficiently than non-preconditioned MSC. IFN-γ-primed MSC inhibited IFN-γ secretion from NK cells, being partially mediated by IDO and prostaglandin-E2 (PGE-2). Additionally, preconditioning with IFN-γ increased the expression of class I HLA molecules and reduced the expression of the activating ligand NKG2D on the surface of MSC, decreasing their susceptibility to NK cytotoxicity [21].

In comparative proteomic analyses of human bone marrow-derived MSC (BM-MSC) primed with IFN-γ, 210 proteins with significantly altered expressions were identified, 169 of which were overexpressed (for example IDO, PDL-1, ICAM-1, VCAM-1, and BST-2) and 41 downregulated (for example ANTXR1, APCDD1L, NPR3, FADS2) [22].

Vigo and coworkers reported that immunosuppressive properties of murine MSC primed with IFN-γ were related to early phosphorylation of signal transducer and activator of transcription (STAT1/STAT3), as well as inhibition of mTOR activity, which leads to the upregulation of genes associated with immunoregulation and downregulation of genes related to differentiation, proliferation, and stemness. In human MSC, the inhibition of the mTOR pathway also enhances their immunoregulatory potential [23].

Notably, IFN-γ-preconditioning prior to MSC cryopreservation was able to improve the immunosuppressive properties after thawing. Similarly with fresh-MSC, thawed IFN-γ-preconditioned MSC were capable of inhibiting T cell proliferation and cytotoxic T cell degranulation via IDO secretion [24]. Moreover, thawed IFN-γ-preconditioned MSC presented lower susceptibility to host T cell cytolysis, compared to untreated MSC. However, the preconditioning of MSC with IFN-γ was not able to revert the homing defect post thaw [24]. The same group has recently shown that IFN-γ-preconditioning can re-establish immunosuppressive properties of senescent MSC by the activation of regulatory molecules, such as IDO. Preconditioning with IFN-γ did not upregulate class-II HLA (HLA-DR) molecules on the surface of senescent MSC, which occurred on early passage MSC [25].

In experimental colitis, human IFN-γ-primed MSC showed higher migration rates to inflammatory sites and a significant reduction of mucosal damage and inflammatory responses, compared with non-primed MSC [26]. On the contrary, Burand and coworkers have recently shown that infusion of thawed IFN-γ-primed human MSC failed to ameliorate a murine model of retinal disease [27]. Noteworthy, several studies have demonstrated that priming with IFN-γ or other inflammatory cytokines leads to upregulation of class I and class II HLA molecules, which makes them more immunogenic and therefore more susceptible to recognition by host immune cells, and subsequently, there is rapid clearance in vivo following administration, especially in xenogeneic transplantation settings [28].

### TNF-α priming

TNF-α priming promotes upregulation of immunoregulatory factors on MSC, such as PGE2, IDO, and HGF, however much less pronounced compared with IFN-γ priming [29]. As an alternative, the combination of inflammatory cytokines for priming MSC may lead to additional effects. In this context, preconditioning with both TNF-α and IFN-γ is capable to increase factor H production by MSC, which potently inhibits complement activation in dose- and time-dependent manners. Factor H production by MSC is significantly suppressed by the inhibition of PGE2 and IDO [30].

François and colleagues have demonstrated that IFN-γ and TNF-α priming increases IDO activity in MSC, which results in monocyte differentiation into IL-10-secreting M2 immunosuppressive macrophages (CD14^+^/CD206^+^). Those differentiated M2 macrophages were then implicated in the suppression of T cell proliferation via IL-10 secretion, thereby amplifying the immunosuppressive properties of MSC [31].

Priming of murine MSC lines with both IFN-γ and TNF-α enhances their anti-proliferative effects on T cell proliferation assays and involves increased expression of the nitric oxide synthetase 2 (NOS2) gene, which results in enhanced production of nitric oxide which is an important immunosuppressive molecule in murine models [32]. MSC primed with IFN-γ and TNF-α were also capable of inhibiting delayed-type hypersensitivity responses in vivo [23, 32].

The expression of IDO decreases after medium removal and freeze-thawing the MSC products [33]. However, preconditioning of MSC with IFN-γ and TNF-α induces chromatin remodeling in the IDO1 promoter, which correlates with increased H3K9 (histone H3 at lysine 9) acetylation concomitantly with a reduction in trimethylated H3K9. Curiously, these chromatin alterations are maintained even after the cryopreservation process. Upon re-exposure to cytokines, previously IFN-γ-treated MSC are able to quickly accumulate high IDO1 mRNA levels [33].

Nevertheless, MSC from different donors primed with TNF-α and IFN-γ exhibit variable suppressive effects upon T cell proliferation, probably due to variable upregulation of IDO activity [31]. Amati and coworkers have shown significant differences in the inhibitory potential of MSC from different cord blood-derived samples, particularly following priming with TNF-α and IFN-γ [34].

### IL-17 priming

IL-17 is another pro-inflammatory cytokine that has been used for MSC priming in some studies. Increased proliferation of human and murine BM-MSC was demonstrated upon dose-dependent IL-17 priming [35–37]. In human MSC, this effect was dependent on the generation of reactive oxygen species (ROS) from the activation of the adapter molecule ACT-1 and TNF receptor-associated factor 6 (TRAF-6). IL-17 priming was able to induce greater migration, motility, and osteoblastic differentiation of MSC [36]. However, there are some conflicting results in the literature.

Some studies show that IL-17 promotes osteogenic differentiation [36, 38] and inhibits adipogenic differentiation of human MSC by enhancing IL-6 and IL-8 mRNA expression during the differentiation process [39]. In contrast, osteogenic differentiation is suppressed in murine MSC by IL-17 priming via IκB kinase and factor nuclear kappa B (NFκB) [40]. Kondo et al. showed that IL-17 inhibits chondrogenic differentiation of human MSC through suppression of the protein kinase A and consequently decreased phosphorylation of SOX9, a transcription factor involved in chondrogenesis [41]. Another study showed that IL-17 does not affect the differentiation potential of murine BM-MSC [42]. Together, these results suggest that the phenotypic profile, functional heterogeneity, differentiation potential, and the response to inflammatory priming of MSC may be influenced by their origin (species and the tissue source).

Murine BM and adipose tissue (AT)-derived MSC primed with IL-17, TNF-α, and IFN-γ have increased T cell immunosuppressive capacity mediated by inducible nitric oxide synthase (iNOS) production. MSC primed with this synergic combination of cytokines were also able to reduce inflammation and tissue injury in murine model of hepatitis, also by an iNOS-dependent mechanism [43].

Human BM-MSC, preconditioned with IL-17, presented the immunophenotype, morphology, and class I MHC expression levels comparable to untreated MSC. There was no increased expression of class II MHC and co-stimulatory molecules in MSC, therefore maintaining their hypoimmunogenic phenotype [﻿44﻿]. Functional studies showed that IL-17-primed MSC presented higher immunosuppressive potential upon proliferating T cells, which was attributed not to IDO, cyclooxygenase-2 (COX-2), or TGF-β, but to increased IL-6 expression. In this study, IL-17-primed MSC inhibited T cell secretion of Th1 cytokines (TNF-α, IFN-γ, and IL-2) and promoted the generation of iTreg cells [44]. Subsequently, 67 differentially expressed genes mainly associated with migration and chemostatic responses (such as MMP1, MMP13, and CXCL6) were identified between IL-17-primed and untreated MSC [44].

### Other combinatory strategies of pro-inflammatory cytokines

MSC from distinct sources might respond differently to priming with combinations of pro-inflammatory factors [45]. Pro-inflammatory cytokine cocktails markedly induced the expression of immunoregulatory molecules and cell-adhesion proteins in MSC from different tissue sources. Notably, only BM-MSC presented a slight increase in HLA-DR expression. In addition, WJ-MSC constitutively produce the highest levels of HGF, which are increased after priming with pro-inflammatory cytokine cocktail [45].

MSC derived from AT, BM, foreskin, or Wharton’s jelly, primed with an pro-inflammatory cytokine cocktail (IL-1β, TNF-α, IFN-α, and IFN-γ), presented different expression levels of the immunoregulatory genes FGL2, GAL, SEMA4D, SEMA7A, and IDO1 [46, 47]. In primed foreskin-derived MSC, 16 miRNAs were differentially expressed, 13 of which were downregulated (miR-27a, miR-145, miR-149, miR-194, miR-199a, miR-221, miR-328, miR-345, miR-423-5p, miR-485-3p, miR-485-5p, miR-615-5p, and miR-758) and 3 were upregulated (miR-155, miR-363, and miR-886-3p). These miRNAs are important to regulate immune responses and have already been related to the differentiation potential and immunomodulatory function of MSC [46].

Human MSC treated with a combination of the pro-inflammatory cytokines IL-1β, IL-6, and IL-23 displayed similar morphology, immunophenotype, co-stimulatory molecule expression profiling (except for up-regulation of CD45), and suppressive ability of allogeneic T cell proliferation, compared with untreated MSC. These primed MSC produced higher levels of TGF-β and lower levels of IL-4 than untreated MSC. The authors suggested that pro-inflammatory cytokines upregulate MSC efficacy for therapy of inflammatory and autoimmune disorders [48].

LPS/TNF-α-primed murine MSC have induced in vitro polarization of M2 macrophage (immunomodulatory macrophage profile, expressing Arg1^high^ and CD206^high^), compared with untreated MSC. Increased PGE2 secretion was associated with high expression of arginase-1 by LPS/TNF-α-primed murine MSC [49]. Moreover, LPS/TNF-α-primed MSC showed increased alkaline phosphate activity and bone mineralization capacity [49, 50].

Global transcriptome profiling of human BM-MSC primed with IL-1β revealed upregulation of genes related to several biological processes linked to NF-κB pathway, such as cell survival, migration and adhesion, chemokine production, induction of angiogenesis, and modulation of the immune response [51]. Additionally, preconditioning of umbilical cord-derived MSC (UC-MSC) with IL-1β resulted in increased immunosuppressive capacities and migration ability to inflammatory sites, being sufficient to attenuate the development of murine colitis. Moreover, IL-1β-primed MSC upregulated the expression of CXCR4, COX-2, IL-6, and IL-8 genes, and the infusion of these cells led to the polarization of peritoneal M2 macrophages, increased frequencies of Treg and Th2 cells, and decreased percentage of Th1 and Th17 cells in the spleen and mesenteric lymph nodes [52].

Preconditioning of MSC from different donors with IL-1α or IL-1β induced high secretion of granulocyte-colony stimulating factor (G-CSF). Conditioned media of primed MSC induced a stronger response of immortalised mouse microglial BV2 cells in the presence of bacterial LPS, evidenced by decreased secretion of inflammatory mediators, such as IL-6 and TNF-a, and increased production of IL-10, an important immunoregulatory cytokine. These results, therefore, support the potential use of pre-conditioning of MSC in future therapies for inflammatory disorders [53].

UC-MSC primed with TGF-β1 displayed increased proliferation and marked upregulation of extracellular matrix genes, notably fibronectin. TGF-β1-primed MSC survived longer in damaged lungs and reduced the severity of lipopolysaccharide-induced lung injury [54]. Additionally, some studies have demonstrated that TGF-β1 is capable of inducing the mobilization and migration of BM-MSC to bone remodeling. N-cadherin-mediated intercellular interactions and noncanonical signaling molecules activated through TGF-β type I receptor, such as Akt, extracellular signal-regulated kinase 1/2 (ERK1/2), focal adhesion kinase (FAK), and p38, are required to increase the migration capacity of MSC [55].

Besides cytokines, growth factors have been explored for preconditioning of MSC, aiming to improve their properties and therapeutic efficacy. Dental pulp-derived MSC (DP-MSC) preconditioned with fibroblast growth factor-2 (FGF-2) exhibited higher angiogenic potential by secretion of vascular endothelial growth factor (VEGF) and HGF, compared with hypoxia conditioning. In addition, FGF-2 priming increased the number of cells expressing Stro-1 and CD146 progenitor markers on MSC cultures [56].

To summarize, many pro-inflammatory cytokines or growth factors have been used for priming of MSC isolated from different species and/or tissues. As a general effect, pro-inflammatory priming increases the immunosuppressive properties of MSC by stimulating the secretion of anti-inflammatory and immunomodulatory factors. Three disadvantages can be pointed out, which are the increased immunogenicity of MSC after priming, high costs of priming with recombinant cytokines, and variable response of MSC from different sources upon pro-inflammatory priming.

## MSC priming with hypoxia

Oxygen availability in the tissues depends on vascularization and metabolic activity and is much lower than in the environment or under normoxic cell culture conditions (20–21%). Oxygen availability in the bone marrow, as an example, can range from 1 to 7%. In neonatal tissues such as the placenta and UC, considered highly promising sources of MSC due to superior plasticity and shorter doubling time, oxygen tension rarely exceeds 5%. Cells in these hypoxic tissues are already physiologically adapted to these conditions.

In this context, MSC expansion in environments with high levels of oxygen may lead to cellular stress. Later, when transplanted in vivo, these ex vivo expanded cells must re-adapt to the new environment [57]. High environmental levels of O_2_ can lead to early senescence, extension of population doubling time, and DNA damage [58–61].

Several studies have already demonstrated that MSC cultured under hypoxic conditions have increased proliferation and secrete various soluble bioactive factors [18, 62]. Under hypoxic conditions, MSC also have high angiogenic and regenerative potentials [63] as well as extended survival in damaged tissues after transplantation [64]. (Table 2, Fig. 2).

**Table 2:** Priming of MSC with hypoxia

Stimuli	Source MSC	Model/disease	In vivo*/*in vitro	Results	References
5% O2	Umbilical cord	Acute spinal cord injury model	In vitro*/*in vivo (rats)	Increased migration, engraftment, and survival; increased tissue preservation and axonal regeneration.	[66]
5% O2	Wharton’s jelly	–	In vitro	Conditioned-medium increased migration and tube formation in vitro, partially reduced by prior inhibition autophagy.	[74]
2.5% O2	Bone marrow	Radiation-induced lung injury model	In vitro*/*in vivo (mice)	Upregulated HIF-1α, increased survival and the antioxidant ability, increased efficiency in the treatment of radiation-induced lung injury.	[79]
2–2.5% O2	Placenta	–	In vitro	Upregulated glucose transporters, adhesion molecules and increased angiogenic potential.	[67]
2% O2	Adipose tissue	Murine hindlimb ischemia model	In vitro*/*in vivo (mice)	Enhanced proliferation, survival, and angiogenic cytokine secretion in vivo*.*	[68]
1.5% O2	Bone marrow	Bleomycin-induced pulmonary fibrosis model	In vitro*/*in vivo (mice)	Improved pulmonary functions and reduced inflammatory and fibrotic mediators in vivo*.*	[78]
1% O2	Human cord blood	–	In vitro	Increased the survival and pro-angiogenic capacity in ischemia-like environment, induced anti-apoptotic mechanisms, and increased VEGF secretion.	[65]
1% O2	Bone marrow	Intramuscular injection into immune-deficient mice	In vitro*/*in vivo (mice)	Reduced cell death under serum-deprivation conditions, decreased cytochrome c and HO-1 levels, enhanced survival in vivo.	[77]

UC-MSC cultured under hypoxic conditions usually adapt to reduced oxygen consumption and demonstrate greater proliferative capacity; reduced or absent cellular apoptosis rates; induction of HIF-1α (hypoxia-induced factor-1 alpha); elevation of PDK-1, GLUT-1, and LDH gene expression (associated with energy metabolism); and reduction of mitochondrial respiration [57]. Consequently, hypoxia-primed MSC present increased glucose consumption, lower reactive oxygen species production, lower telomeric shortening rates, and decreased cellular senescence [57].

Interestingly, increased HIF-1α expression associates with high expression of chemokine receptors in MSC, such as CXCR4, CXCR7, and CX3CR1, which are involved in the trafficking and homing of transplanted MSC to target tissues [59].

Exposure of MSC to hypoxia induces anti-apoptotic mechanisms through phosphorylation of AKT and BAD, increased expression of BCL-XL and BAG-1, reduced caspase-3/7 activity, lactate dehydrogenase (LDH) release, and increased VEGF secretion [65].

Zhilai and coworkers showed that hypoxia-preconditioned UC-MSC produce higher levels of VEGF, HGF, and brain-derived neurotrophic factor (BDNF). In addition, in a rat model of spinal cord injury, transplantation of hypoxia-preconditioned UC-MSC led to higher migration potential and engraftment, increased cell survival, reduced apoptosis, and inflammatory infiltration, which were associated with axonal regeneration and improved tissue function [66].

Placenta-derived MSC primed with hypoxia showed increased expression of adhesion molecules, including fibronectin 1 (FN1), E-cadherin (CDH1), N-cadherin (N-CAD or CDH2), and integrins (ITGA2, ITGA5, ITGB1, ITGB3, ITGB5) required for angiogenesis. In addition, they presented increased in vitro angiogenic potential, expression of glucose transporters (GLUT-1, GLUT-2, GLUT-3), and insulin secretion upon glucose stimulation, which are vital for the wound healing process [67].

Hypoxia induces upregulation of GRP78 (78-kDa glucose-regulated protein) through increased HIF-1α expression in human AT-MSC [68]. Subsequently, hypoxia-induced GRP78 regulates Akt, mTOR, and p70S6k phosphorylation, indicating that hypoxic preconditioning enhances MSC proliferation and migration through the Akt signaling pathway. This effect has been demonstrated in a murine hindlimb ischemia model, through regulation of stress- and apoptosis-associated proteins and increased secretion of angiogenic factors such as hVEGF, hHGF, and hFGF [68] (Table 2).

The expression pattern of miRNA is related to MSC fate [69, 70]. In this context, hypoxia preconditioning induced early miR-675-5p upregulation and subsequent angiogenic response by increasing VEGF secretion and VEGF receptor 1 (VEGFR-1/FLT-1) expression. In addition, miR-675-5p inhibition caused a reduction of HIF1A gene expression and upregulation of HIF1A negative regulators, suggesting its role in the stabilization of HIF1A. Overexpression of miR-675-5p caused downregulation of stemness markers (CD44, CD90, and CD73) and promoted expansion of the chondro-osteoblast precursor markers in vitro [71].

Gonzalez-King and colleagues have demonstrated that HIF-1α-overexpressing MSC secrete higher amounts of exosomes compared to control MSC. These exosomes show overexpressed miRNA content, including miR-15, miR-16, miR-17, miR-31, miR-126, miR-145, miR-221, miR-222, miR-320a, miR-424, and Notch pathway-related proteins [72]. Their results indicate that exosomes derived from MSC overexpressing HIF-1α have increased angiogenic capacity and could be applied to the treatment of ischemia-related diseases [72].

Hypoxic priming increases HIF-1α-dependent autophagy in MSC derived from bone marrow and Wharton’s jelly [73, 74]. Conditioned medium derived from WJ-MSC primed with hypoxia led to enlarged migration and tube formation from HUVECs. The angiogenic potential and secreted levels of angiogenin and VEGF were reduced by autophagy inhibition [74].

Preconditioning of AT-MSC with hypoxia and IFN-γ led to synergic effects [75]. The authors reported increased anti-proliferative capacity that correlated with higher secretion of IDO and HLA-G, and significant upregulation of proteins involved in gluconeogenesis [75]. Dual priming with hypoxia/IFN-γ decreased the production of VEGF and IL-8, increased the secretion of MCP-1 and IL-6, and increased endothelial cell migration in a “wound closure” assay [76].

The properties of hypoxia-primed-MSC have also been shown in murine disease models. Hypoxic-primed MSC showed metabolic alterations and enhanced survival after intramuscular injection into immune-deficient mice [77].

Hypoxia-preconditioned murine MSC demonstrated upregulation of pro-survival genes, with enhanced survival of engrafted cells, and increased secretion of anti-apoptotic, anti-oxidant, and growth factors. Bleomycin-induced pulmonary fibrotic mice treated with hypoxia-preconditioned MSC showed improved pulmonary functions and reduced inflammation and fibrosis [78].

Similarly, hypoxic-preconditioned MSC showed increased cell viability, enhanced proliferation potential, decreased ROS production, and increased antioxidant GSH and SOD levels. Moreover, they presented higher HIF-1α and Akt expression levels, important in the resistance to hypoxia and ROS stress, which are essential in the transplantation setting [79]. In the murine model of radiation-induced lung injury, infusion of hypoxic-preconditioned MSC alleviated both early radiation-induced pneumonia and late pulmonary fibrosis. Notably, hypoxia-primed MSC displayed a more pronounced therapeutic effect compared to normoxia MSC [79].

In conclusion, hypoxia priming has been used to mimic the in vivo MSC niche conditions, aiming to improve the therapeutic efficacy of MSC from different species and/or tissues. In general, hypoxia priming greatly alters cell metabolism during expansion, increases resistance to oxidative stress, and thereby improves the engraftment, survival in ischemic microenvironments, and angiogenic potential of transplanted MSC.

## MSC priming with pharmacological or chemical agents

Priming with pharmacological or chemical agents is a promising strategy to improve MSC engraftment and survival in damaged tissues and consequently the therapeutic efficacy [14]. Table 3 and Fig. 2 summarize some of these approaches to improve MSC-based therapies for different diseases and/or applications.

**Table 3:** Priming of MSC with pharmacological drugs and other chemical agents

Stimuli	Source	Model/disease	In vitro*/*in vivo	Results	Reference
VPA and SP1	Umbilical cord	–	In vitro	Increased proliferation; improved anti-inflammatory activities.	[80]
VPA	Bone marrow (murine)	Huntington’s disease model	In vivo (mice)	Reduced neuropathological features.	[81]
5-aza-dC	Vascular endothelial cells (VECs) derived from bone marrow	Trans-differentiation angiogenesis	In vitro (Matrigel)	Increased endothelial markers expression; improved angiogenesis capacity on Matrigel.	[82]
DFO	Bone marrow	–	In vitro	Reduced mitochondrial oxygen consumption and apoptosis, up-regulated glycolysis and survival-related genes.	[87]
DNP	Bone marrow	Myocardial infarction model	In vitro*/*in vivo (rats)	Increased expression of cardiomyogenic factors (GATA-4, Nkx2.5, connexin-43, and atrial natriuretic peptide (ANP); increased expression of genes involved in adhesion and homing; increased expression of VEGF and HIF; improved cardiac function and reduced scar formation.	[90]
DMOG	Bone marrow	Ischemic heart model	In vitro*/*in vivo (rats)	Upregulated survival and angiogenic factors (HIF-1α, VEGF, Glut-1); reduced cell death; enhanced angiogenic activities; decreased infarct size.	[91]
ISO	Bone marrow	Stroke model	In vitro*/*in vivo (rats)	Upregulated CXCR4 and HIF-1α expression; improved engraftment into the ischemic brain and improved functional recovery.	[92]
CCPA	Dental pulp	Osteogenesis	In vitro	Improved proliferation and osteogenic differentiation; upregulated RUNX-2 and alkaline phosphatase expression; improved mineralization in extracellular matrix.	[96]
ATRA	Bone marrow (rat)	Excisional wounds model	In vitro*/*in vivo (rats)	Upregulated COX-2, HIF-1, CXCR4, CCR2, VEGF, Ang-2 and Ang-4 gene expression; improved wound healing.	[100]
ATRA	Bone marrow (murine)	Emphysema model	In vivo (mice)	Increased MSCs survival in the lungs; improved airway function.	[101]
ATRA	Bone marrow	Ankylosing spondylitis model	In vitro	Decreased secretion of inflammatory cytokines TNF-α, IL-17A and IFN-γ; increased IL-6 secretion; induced Treg.	[102]
Rapamycin (short exposure)	Bone marrow	–	In vitro	Upregulated COX-2/PGE2; decreased PBMCs and splenocytes proliferation.	[99]

*VPA* valproic acid, S*P1* sphingosine-1-phosphate, *5-aza-dC* 5-aza-2′-deoxycytidine, *DFO* desferrioxamine, *DNP* 2,4-dinitrophenol, *DMOG* dimethyloxalylglycine, *ISO* isoflurane, *CCPA* 2-chloro-N6-cyclopentyl-adenosine, *TGF-β1* transforming growth factor β1, *IGF* insulin growth factor, *ATRA* all-trans retinoic acid

MSC homing and engraftment are regulated by the interaction between stromal-derived factor 1 (SDF-1) and CXCR4 receptor [1]. In this context, low doses of histone deacetylase inhibitor valproic acid (VPA) and bioactive lipid sphingosine-1-phosphate (S1P) are capable of activating UC-MSC. Thus, VPA/S1P priming improves MSC migratory activity in response to SDF-1, concomitant with the activation of both MAPKp42/44 and AKT signaling [80]. Moreover, MSC priming with VPA/S1P also augmented proliferation and anti-inflammatory activities [80]. In an experimental model of Huntington’s disease, priming of MSC with VPA and lithium, prior to intranasal delivery, enhanced the biological potential and therapeutic properties of MSC, evidenced by reduced neuropathological features and functional improvement [81].

Good vascularization remains an obstacle to proper tissue regeneration. Disorders such as spinal cord injury, cerebral ischemic disease, myocardial infarction, and diabetes mellitus are characterized by endothelial injury or dysfunction, impairing blood flow to injured areas. Vascular endothelial cells (VECs) derived from transplanted MSC may be used as an important therapeutic strategy. DNA methylation is involved in MSC differentiation in endothelial cells. The DNA methyltransferase inhibitor, 5-aza-2′-deoxycytidine (5-aza-dC), is able to induce differentiation of MSC in VECs in vitro by increasing the expression of endothelial markers and their angiogenic capacity [82].

Cardiovascular diseases (CDs), including myocardial infarction and ischemic heart diseases, are a major health problem worldwide [83]. Despite controversial results, MSC administration in patients with CDs promotes angiogenesis and myogenesis, restores cardiac stem cell niches, reduces infarcted area and scar formation, and improves cardiac function [64, 84–86]. The major problem of MSC-based therapies for CD is the low cell survival in the infarcted and ischemic areas [64]. In this context, preconditioning strategies may increase the survival of transplanted MSC in vivo.

As previously discussed, hypoxic-primed MSC have increased survival in damaged tissues after transplantation [64]. In this context, some studies have evaluated hypoxia-mimetizing chemical reagents for priming MSC. A recent study analyzed the influence of desferrioxamine (DFO) on BM-MSC proliferation, apoptosis, and metabolic changes. DFO is a hypoxia-mimetic reagent that decreases the activity of prolyl-hydroxylases and consequently inhibits HIF-1α hydroxylation. In low concentrations, DFO decreased mitochondrial activity and apoptosis and upregulated the expression of genes associated with glycolysis, including hexo-kinase 2 (HK2), pyruvate dehydrogenase kinase 1 (PDK1), BCL2-interacting protein 3 (BNIP3), lactate dehydrogenase A (LDHA), and VEGF, as well as genes related to cell viability and survival. The inhibitory effect of DFO upon MSC proliferation was associated with suppression of nucleic acid metabolism by reducing ribonucleotide reductase activity. The DFO-primed MSC also showed decreased adipogenic and osteogenic differentiation [87]; therefore, it is not suitable for the treatment of bone diseases.

Chemicals that inhibit the electron transportation chain, such as 2,4-dinitrophenol (DNP) [88], hypoxia-inducible factors (HIF-1, HIF-1α, HIF-1β), or prolyl-hydroxylase inhibitor dimethyloxalylglycine (DMOG) [89], also induce hypoxic environments. Preconditioning with DNP induces upregulation of adhesion and homing genes in MSC, as well as increased secretion of cardiomyogenic factors. Intramyocardial injection of DNP-conditioned MSC in infarcted rats showed good cellular engraftment and significant improvement of cardiac function, reduction of scar formation, improved angiogenesis, and maintenance of left ventricular wall thickness [90]. Similarly, MSC preconditioning with DMOG upregulates the expression of hypoxia survival and angiogenic factors, including HIF-1α and VEGF [91]. In a rat model of the ischemic heart, DMOG-primed MSC intramyocardially transplanted into peri-infarcted regions showed higher survival rates, enhanced angiogenesis, and improved heart function, compared with non-primed MSC [91].

The volatile anesthetic isoflurane (ISO) can be cytoprotective for MSC. In vitro preconditioning of BM-MSC with ISO (low doses and short treatment duration, 2% ISO for 4 h) increases cell viability and migration potential. There was hypoxia-induced upregulation of CXCR4 and HIF-1α, whereas no effect was observed in the expression of HIF-1β [92]. However, longer exposures to ISO at higher doses decreased MSC viability and migration potential. In a rat model of stroke, preconditioned ISO-MSC improved the function and engraftment into the ischemic brain [92].

In addition, MSC-based therapy has emerged as an option for bone and tendon tissue engineering and regeneration [93, 94]. Therefore, priming of MSC to improve engraftment and osteogenic/chondrogenic differentiation potentials may be convenient for the treatment of bone and tendon disorders. In this context, adenine-based purines may be used as biochemical priming factors/agents. The adenine-based purines, including nucleosides or adenosine, are ubiquitous substances released from several cells types and are able to interact with the adenosine receptor (AR) family, which regulates many physiological/pathological processes [95]. The selective agonist 15–60 nM 2-chloro-N6-cyclopentyl-adenosine (CCPA) interacts with the adenosine A1 receptor (A1R), thereby increasing the proliferation of human DP-MSC by activating WNT signaling. As well as this, CCPA enhances MSC osteogenic differentiation potential by the upregulation of the RUNX-2 and ALP expressions. Furthermore, extracellular matrix mineralization is improved by preconditioning of MSC with CCPA [96].

The source of cells also plays an important role in cell fate after priming. A1R expression is higher in AT-derived MSC than in DP-MSC. Accordingly, AT-MSC primed by CCPA substantially increased their osteogenic differentiation and titanium scaffold colonization. Thus, AT-MSC primed with CCPA could be advantageously used in regenerative orthopedics [97].

Considering that tenogenesis is a complex process, tendon repair is a clinical challenge [98]. The AKT-mTOR axis is involved in the synthesis of important proteins for tenocyte differentiation and tendon homeostasis, including type I collagen (Col-I) and other extracellular matrix proteins. In experimental models, mTOR depletion has been associated with tendon defects, and MSC priming with protenogenic growth factor (PGF), TGF-β1, and insulin growth factor (IGF) upregulates mTOR expression and signaling. On the other hand, statin treatment abrogates mTOR signaling and reduces the expression of Col-I and tenascin during in vitro MSC tenogenesis and in injured tendon tissues [98]. In conclusion, the increase of the AKT-mTOR axis signaling in MSC, by specific priming, leads to improved tendon differentiation and may constitute a novel therapeutic approach to tendinopathies and tendon injuries (Table 3).

The inhibition of the mTOR-signaling pathway by rapamycin has different effects on MSC. Short exposure of BM-MSC to rapamycin, just before coculturing with activated human CD4^+^ T cells or mouse splenocytes, decreased mTOR signaling and enhances their in vitro immunosuppressive properties by COX-2 and PGE2 upregulation, independently of the inflammatory stimulus. Conversely, such effects were not observed after prolonged exposure of MSC to rapamycin [99].

All-trans retinoic acid (ATRA) binds to nuclear retinoic acid receptors (RARs) and is also used as a priming factor. ATRA plays critical roles in cell growth, including differentiation, apoptosis, and immune function. Preconditioning of rat BM-MSC with ATRA upregulates COX-2, HIF-1, CXCR4, CCR2, VEGF, Ang-2, and Ang-4 gene expressions. In vivo, ATRA-primed MSC enhanced wound healing potential [100]. Priming of mouse BM-MSC with ATRA has demonstrated significant therapeutic benefits in an experimental emphysema model. In this work, ATRA led to the activation of p70S6k1, thereby improving lung tissue repair and MSC survival [101].

In addition, human BM-MSC primed with ATRA, when cocultured in vitro with activated peripheral blood mononuclear cells from ankylosing spondylitis (AS) patients, secreted high levels of IL-6 and induced the expansion of Treg subsets. Moreover, ATRA-primed MSC modulated the inflammatory cytokine profile of PBMC from AS patients, reducing the secretion of TNF-α, IL-17A, and IFN-γ [102] (Table 3).

Taken together, these studies demonstrate potentially beneficial effects of MSC priming with pharmacological drugs or chemical agents. Although their effects vary greatly, most stimuli improve MSC survival and increase their differentiation potential and regenerative and/or immunomodulatory properties.

## MSC priming with biomaterials or different culture conditions

Several studies have demonstrated that the modulation of the biochemical and biophysical microenvironment may influence MSC fate and also enhance their therapeutic potential [12, 103, 104]. Strategies to modulate the environment include the use of specific biomaterials for tissue engineering applications, three-dimensional (3D) MSC cultures, and cell treatment with different culture conditions (lineage-specific and conditioned culture medium), among others (Table 4, and Fig. 2).

**Table 4:** Priming of MSC with biomaterials or different culture conditions

Stimuli	MSC source	Model/disease	In vitro*/*in vivo	Results	References
3D cell culture in collagen-hydrogel scaffold	Umbilical Cord	–	In vitro	Induced chondrogenesis differentiation by increasing expressions of collagen II, aggrecan, COMPS.	[118]
3D cell culture in chitosan scaffold	Bone marrow (rat)	–	In vitro	Induced chondrogenesis differentiation by increased production of collagen type II.	[119]
3D cell culture of composite combining an affinity peptide sequence (E7) and hydrogel	Bone marrow (rat)	–	In vitro	Increased cell survival, matrix production, and improved chondrogenic differentiation ability.	[120]
3D cell culture of alginate/chondroitin sulfate	Bone marrow	–	In vitro	Induced type II collagen synthesis and chondrogenesis in the scaffolds.	[104]
3D cell culture of collagen/hydroxyapatite, hydroxyapatite, and biphasic calcium phosphate	Bone marrow (rat)	–	In vitro	Exhibited the highest osteogenic capacity in collagen/hydroxyapatite, but the poorest in hydroxyapatite.	[123]
3D cell culture in poly(ethylene glycol)-variant scaffolds	Bone marrow	–	In vitro	Upregulated osteogenic markers and osteocalcin expression.	[125]
3D cell culture of mineralized collagen sponges and alpha-tricalcium phosphate (alpha-TCP)	Bone marrow	–	In vitro	Improved seeding efficacy and increased osteogenic marker genes (mineralized collagen scaffold).	[126]
3D cell culture in hydrogel	Bone marrow (murine)	Excisional wound healing model	In vitro*/*in vivo (mice)	Induced angiogenic cytokines and expression of Oct4, Sox2, Klf4 in vitro and enhanced wound healing in vivo*.*	[129]
Encapsulation in hydrogel	Bone marrow (rat)	Diabetic ulcers model	In vitro*/*In vivo (rats)	Promoted granulation tissue formation, angiogenesis, extracellular matrix secretion, wound contraction, and re-epithelialization.	[130]
Glucose concentration in the culture medium	Telomerase-immortalized (hMSC-TERT)	–	In vitro	High-glucose concentration (25 mM) increased proliferation and osteogenic differentiation.	[132]
High glucose concentration in the culture medium	Bone marrow		In vitro	Decreased chondrogenic capacity.	[133]
Medium from cardiomyocytes exposed to oxidative stress and high glucose	Bone marrow (diabetic mouse)	Diabetes induced with streptozotocin model	In vitro*/*in vivo (mice)	Enhanced survival, proliferation and angiogenic ability, increased the ability to improve function in a diabetic heart.	[134]
Spheroid formation (different techniques)	Bone marrow		In vitro	Enhanced homogenous cellular aggregates formation and improved osteogenic differentiation (low attachment plates).	[139]
Spheroids formation (hanging-drop)	Bone marrow	Zymosan-induced peritonitis model	In vitro*/*in vivo (mice)	Expressed high levels of anti-inflammatory (TSG-6 and STC-1) and anti-tumorigenic molecules compared to 2D culture, suppressed inflammation in vivo.	[140]
Spheroid formation (chitosan films)	Adipose tissue	Cutaneous wound model	In vitro*/*in vivo (mice)	Increased expansion efficiency with less senescence and enhanced migration; improved healing and enhanced angiogenesis in the wounds.	[142]
Spheroids formation (hanging drop)	Cord blood	Hindlimb ischemia model	In vitro*/*in vivo (mice)	Improved engraftment; increased the number of microvessels and smooth muscle α-actin-positive vessels.	[143]

ECM is an important component of the bone marrow niche, acting as a physical support for cell adhesion in a 3D environment. In order to mimic the MSC niche, researchers have explored the use of different 3D biomaterials for cell culture [11]. In fact, MSC applications in tissue engineering are largely associated with the development of biomaterials, which can direct MSC fate towards the desired phenotype and also provide a microenvironment that allows for structural and biochemical cellular support, thereby enhancing tissue healing [105]. Moreover, biomaterial-based approaches could protect cells from death due to anoikis and/or inflammation, facilitating their homing [14]. MSC can be explored for many tissue-engineering therapies in the repair of cartilage, bone, cardiac, and/or skeletal defects [106–110] (Table 4).

The ideal biomaterial for cell culture should be able to provide reproducibility, biocompatibility, and clinical relevance for a particular disease [111]. Other important aspects to be considered in this setting are the biomaterial characteristics, including stiffness, topography, geometry, and chemical composition [112, 113]. Engler and coworkers [114] described, for the first time, that matrix stiffness influences MSC lineage differentiation. They demonstrated that the elasticity of the matrices used for MSC growth could modulate cell differentiation and result in distinct phenotypes. When cells were cultured in soft substrates, they exhibited a neuronal phenotype. Yet, matrices with intermediate stiffness stimulated myogenic differentiation, and rigid materials induced an osteogenic phenotype [114].

Similarly, biomaterial topography also influences MSC differentiation. Wu and coworkers tested the effects of various nano-topographical patterns to induce MSC towards chondrogenic differentiation. Different material topographies triggered changes in MSC differentiation, underscoring the importance of incorporating topographical design in biomaterials for tissue engineering [103].

Biomaterials can be classified as natural and synthetic matrices. Natural matrices include alginates, collagen, fibrin, chitosanes, gelatine, and hyaluronates, while synthetic scaffolds include bioresorbable polymers, such as polylactic acid (PLA) and polyglycolic acid (PGA), polyethylene, and polypropylene. Biomaterials can be modified to improve cellular activities and functionality by incorporating functional groups, side chains, chemotactic factors, or matrix proteins [115]. For example, by immobilizing RGD (Arg–Gly–Asp) motifs in alginate microspheres, researchers were able to enhance MSC attachment, growth, and angiogenesis [116].

Cartilage defects due to age, osteoarthritis, trauma, and developmental disorders may cause joint pain and loss of mobility [117]. MSC in culture can be induced to differentiate into chondrocytes in polymeric scaffolds, such as alginate, collagen-hydrogel, hyaluronan, and chitosan [104, 118–120].

MSC have become one of the most promising cell types for bone tissue engineering applications. Accordingly, bone has become the second most frequently transplanted tissue in the world [121]. The combination of distinct reagents for culture medium supplementation and biomaterials successfully induces MSC differentiation into osteoblasts. MSC culture with dexamethasone, ascorbic acid 2-phosphate (AsAP), and β-glycerophosphate resulted in calcium matrix deposition and the expression of late osteogenesis markers [122].

The most used biomaterials for this purpose are hydroxyapatite [123], poly-l-lactic-acid (PLLA) [124], tyrosine-derived polycarbonates copolymerized with poly(ethylene glycol) (PEG) [125], and mineralized collagen [126] (Table 4).

Hydrogels are an ideal physicochemical mimetic of natural ECM. MSC suspension and/or encapsulation in hydrogel matrices could improve cell viability and survival. The viscosity of hydrogel reduces mechanical forces applied in the syringe-based administration, significantly diminishing MSC loss during the procedure [14]. In addition, MSC encapsulation/culture in hydrogel improves osteogenic [127] and chondrogenic [128] differentiation, accelerates normal wound healing, and promotes neovascularization [129, 130], cell viability, homing, and proliferation [12, 131].

Since the behavior of MSC is strongly regulated by the environment to which they are exposed, the composition of culture medium could be modified and adapted to achieve the desired MSC phenotype, differentiation, and/or therapeutic potential.

Glucose is the main source used by cells to generate ATP, and it has been demonstrated that the glucose concentration in the culture medium results in altered MSC properties, specifically impacting multilineage differentiation. Li and coworkers showed that MSC exposed to high glucose medium (25 mM) reduced the colony forming activity and induced premature senescence [132]. On the other hand, the osteogenic potential of MSC was higher when the cells were cultured with high-glucose medium compared with low-glucose medium [11]. Tsai et al. also found that high-glucose medium for MSC expansion decreases the chondrogenic capacity by modulation of protein kinase C (PKC) and TGF-β signaling molecules [133]**.**

Khan and coworkers used a different approach based on the use of conditioned medium from cardiomyocytes exposed to oxidative stress and high glucose. MSC cultured in this medium presented increased survival, proliferation, angiogenic potential, and beneficial therapeutic effects when transplanted into the hearts of diabetic animals [134].

In addition to using biomaterials that drive MSC differentiation towards specific phenotypes, a scaffold-free 3D cell culture could be a promising approach to culture undifferentiated MSC. 3D cultured MSC presented significant differences regarding cellular phenotype and biological response compared to monolayer culture. Significant differences were detected between the cellular phenotype and biological response, when compared to traditional monolayer cultures. MSC functionality depends on physical microenvironment, and experiments using cell aggregates show that cell-cell interactions, as well as cell polarity, are essential [105]. 3D culture, also known as multicellular spheroids, facilitates greater cell-cell contact and interaction of cells with the ECM, mimicking in vivo development and signaling activity, thus improving therapeutic properties of human MSC [135]. Currently, it is well-known that MSC cultured in spheroids have enhanced angiogenic, anti-inflammatory, and regenerative effects with improved cell survival after infusion [136].

The organization of MSC into aggregates can be achieved by low attachment plates, hanging drop method, and stirred systems (e.g., spinner flasks, stirred-tank bioreactors). Suspension culture in ultra-low attachment plates (e.g., Corning® Costar® Ultra-Low Attachment plates) is easy to handle; however, it can produce spheroids with different sizes, compromising reproducibility [137]. Hanging drop cultures allow cells to aggregate by gravity, forming a sphere at the bottom of the drop. The size of the sphere can be controlled by the volume of the drop or by the cell concentration [138]. Efforts have been made to produce spheroids in a scalable and reproducible (size distribution) manner. Stirred cultures (stirred tank bioreactor/spinner flasks) employ constant agitation to minimize cellular attachment to the flask surface, creating more homogeneous spheroids in a large scale. Hildebrandt and coworkers showed improved nutrient delivery and increased MSC viability using flasks on a rotating platform [139].

Bartosh and coworkers [140] showed that the potent anti-inflammatory effect exerted by MSC spheroids is attributed to higher levels of expression of the TSG-6 (TNF-stimulated gene 6 protein), a protein with both anti-inflammatory and anti-apoptotic effects [140]. MSC in spheroids enhance immunosuppressive effects by upregulating PGE2 and HGF levels [141]. Cheng and coworkers showed that AT-MSC cultured in spheroids expressed significantly higher levels of pluripotency markers (CXCR4, Nanog, Sox2, and Oct4), leading to the conclusion that 3D configuration also increases MSC stemness [142]. In addition, spheroid formation increases survival of MSC after infusion [143], augments tissue regenerative properties, and more importantly, reduces in vitro senescence [136].

In conclusion, these studies have demonstrated that the modulation of the biochemical and biophysical microenvironment indeed influences MSC differentiation potential, phenotype, and their therapeutic potential [12, 103, 104]. These approaches are very promising as the cell engineering field is growing rapidly.

## MSC priming with other molecules

Most of the MSC priming approaches using other molecules are focused on maximizing their therapeutic potential, mostly by boosting defensive/protective cellular mechanisms in order to escape from the detrimental effects of the host (Table 5, Fig. 2).

**Table 5:** Priming of MSC with other molecules

Stimuli	MSC source	Model/disease	In vitro*/*in vivo	Results	References
LL-37	Placenta	–	In vitro	Increased migration and immunosuppressive function; increased expression of IDO, IL-10, TGF-β, IL-6, and IL-1β; increased TLR-3 levels	[154]
S1P or LL-37	Adipose tissue and cord blood	Pulmonary artery hypertension model	In vitro*/*in vivo (rats)	Increased angiogenic potential by upregulation of VEGFA, CXCR4, PDGF, HGF, and Ang-1; improved self-renewal and anti-inflammatory properties; increased density of lung blood vessels (S1P)	[155]
LPS	Adipose tissue	Partial hepatectomy model	In vitro*/*in vivo (mice)	Increased IL-6, TNF-α, HGF, VEGF levels in vitro; enhanced liver regeneration and decreased IL-6 and TNF-α serum levels.	[146]
LPS and poly(I:C)	Bone marrow	–	In vitro	Induced immunosuppressive TLR3-driven phenotype, secretion CCL10, CCL5, IL-4 and IL-10, PGE2 and IDO (Poly(I:C)); Induced pro-inflammatory TLR4-driven phenotype, secretion of IL-6 and IL-8 (LPS).	[145]
DPS30	Bone marrow	–	In vitro	Increased proliferation and immunosuppressive potential; increased TNF-α, IL-8, TGF-β1, VCAM, CD39, CD73 and adenosine levels.	[147]
Curcumin	Adipose tissue (rat)	Myocardial injury model	In vitro*/*in vivo (rats)	Increased viability; reduced fibrosis and promoted neovascularization by upregulation of VEGF2; decreased myocardial apoptosis	[160]
Curcumin	Bone marrow (rat)		In vitro	Increased osteogenesis differentiation; upregulation of HO-1, RUNX2, and osteocalcin.	[161]
Ang1	Bone marrow (rat)	Acute myocardial infarction model	In vitro*/*in vivo (rats)	Increased cell survival due to Akt phosphorylation and increase expression of Bcl-2.	[162]
Apelin-13	Bone marrow (mice)	–	In vitro	Increased proliferation and decreased apoptosis; induced angiogenesis in hypoxic-ischemic condition by VEGF upregulation.	[157]
DHT	Wharton’s jelly	–	In vitro	Increased proliferation; upregulated cell migration and pro-angiogenic factors, such as MMP-9, VEGF, and angiogenin.	[156]
Oxytocin	Bone marrow (diabetic rat)	Myocardial infarction	In vitro*/*in vivo (rats)	Restored secretion of KLF2; increased angiogenesis in vitro; improved cardiac function and reduced fibrosis in vivo.	[159]
Melatonin	Bone marrow (rat)	Osteoporosis and colitis model	In vitro*/*in vivo (rats)	Preserved self-renewal and differentiation capacity after long-term passaging; preserved therapeutic potential of long-term passaged MSC in bone regeneration and immunotherapy in vivo.	[153]
Melatonin	Bone marrow (rat)	Diabetic nephropathy model	In vitro*/*in vivo (rats)	Increased insulin and decreased angiotensin II levels; improved kidney functions.	[151]
Tetrandrine	Bone marrow	Ear skin inflammation model	In vitro*/*in vivo (mice)	Increased PGE-2 expression; decreased production of TNF-α in vivo*.*	[144]
Ro-31-8425	Bone marrow	Ear skin inflammation model	In vitro*/*in vivo (mice)	Increased homing ability and immunosuppressive potential in vivo through CD11a upregulation and strong adhesion to ICAM-1.	[163]

*LL-37* cathelicidin LL-37, *S1P* shingosine-1-phosphate, *LPS* lipopolysaccharide, *poly(I:C*) polyinosinic:polycytidylic acid, *DHT* dihydrotestosterone, *Ang1* angiopoetin-1

Non-selective (or non-specific) priming approaches stimulate wide effector molecules and signaling pathways [144]. Exogenous danger signals, such as Poly(I:C) or LPS, respectively involved in virus or microbial infections, are agonists for Toll-like receptor 3 (TLR3) and Toll-like receptor 4 (TLR4) signaling. Priming with Poly(I:C) directs MSC polarization into an immunosuppressive phenotype through TLR3 activation. However, LPS activates TLR4 signaling, leading MSC into a more pro-inflammatory profile [145].

MSC priming with low-dose LPS increases the secretion of pro-inflammatory cytokines (such as IL-6 and TNF-α) and of regenerative factors (e.g., HGF and VEGF). The conditioned medium from LPS-primed MSC is able to improve liver regeneration and function [146]. Sangiorgi and coworkers demonstrated that TLR4 stimulation by LPS restricted the suppressive ability of MSC by increasing gene expression of IL-1β and IL-6. On the other hand, MSC stimulation with DSP30 induces TLR9 signaling, leading to reduced TNF-α expression, increased TGF-β1 expression, increased percentages of BM-MSC double positive for CD39 and CD73, and adenosine levels. As a consequence, MSC presented higher proliferative and suppressive potentials [147].

Some studies have demonstrated that melatonin priming can induce chondrogenic differentiation of MSC [148–150]. Melatonin-primed MSC greatly improved renal function in the diabetic nephropathy model, which was correlated with autophagy activation [151]. In a skin wound model, the melatonin-primed MSC also enhanced wound healing and showed increased motility by reorganization of the actin cytoskeleton via FAK/paxillin phosphorylation and melatonin receptor 2 [152]. Shuai and collaborators showed that melatonin priming may prevent the dysfunction and therapeutic failure of long-term passaging MSC in experimental osteoporosis or colitis models [153].

MSC priming with LL-37, a host defense peptide member of the cathelicidin family, enhances the expression of TLR3 and immunosuppressive factors IDO, IL-10, and TGF-β, but no effects were observed in T cell proliferation. In another study, LL-37-primed MSC showed increased migration and higher suppressive effect over T cell proliferation [154].

Priming of MSC with LL-37 and bioactive lipid shingosine-1-phosphate (S1P) improved the therapeutic efficacy of MSC in pulmonary artery hypertension model [155]. S1P/LL-37 priming increased the chemotactic and clonogenic activities of MSC. In addition, this dual priming enhances MSC anti-inflammatory potential by reducing the expression of pro-inflammatory genes, such as CCL2, IL-1β, IL-6, and IL-12. Particularly, S1P conditioning reduces TNF-α secretion from LPS-activated macrophages and enhanced angiogenesis by the secretion of VEGFα, CXCR4, PDGF, HGF, and angiopoietin-1 [155].

The hormone dihydrotestosterone (DHT) stimulates MSC proliferation, migration, and tissue engraftment. In addition, DHT-primed MSC contribute to cardiac regeneration [156]. Other studies have explored the effects of Apelin-13 peptide priming on MSC differentiation, proliferation, and survival in ischemic models. Apelin-13 is an endogenous ligand for the angiotensin receptor. Under hypoxic and normoxic conditions, apelin-13 improves MSC survival through anti-apoptotic effects and promotes the angiogenic properties [157, 158]. Apelin also promotes the upregulation of the autophagy mechanism in MSC, which is related to cellular survival [158].

Diabetes mellitus is a common risk factor for cardiac disease in elderly patients. In these patients, diabetes may impair MSC therapeutic effects. The preconditioning with oxytocin, a hypothalamus hormone, recovered the regenerative and angiogenic properties of diabetes-impaired MSC and consequently improved cardiac function and decreased cardiac tissue fibrosis [159].

Curcumin is a natural antioxidant that protects tissues from oxidative stress and stimulates regeneration [160]. In vivo and in vitro approaches using curcumin priming empowered the regenerative potential of MSC by increasing cell viability and retention, enhancing angiogenesis by VEGF secretion, and reducing apoptosis via heme oxygenase 1 (HO-1) and PTEN/Akt/p53 signaling pathway activation [160]. In addition, rat BM-MSC preconditioned with curcumin showed impaired adipogenesis and increased osteogenesis by superexpression of HO-1 [161].

Liu and coworkers demonstrated that Ang1-preconditioning had increased MSC survival and decreased their apoptotic rate in vitro. Ang1 preconditioning induced Akt phosphorylation and Bcl-2 expression and increased Bcl-2/Bax ratio. However, the PI3K/Akt pathway inhibitor, LY294002, abrogated the protective effect of Ang1 preconditioning. After transplantation, animals treated with Ang1-preconditioned-MSC had lower death rate, reduced infarct size, and better functional heart recovery compared to those treated with non-preconditioned-MSC. Therefore, Ang1-preconditioning of MSC enhanced in vivo survival and heart function after treatment [162].

In contrast with non-selective approaches, selective strategies aim to stimulate few signal-transduction modulators or single pathways [144]. Selective priming with the small peptide tetrandrine stimulates the NF-κB/COX-2 pathway, thereby augmenting PGE2 secretion and consequently boosting the immunosuppressive effects of MSC over activated macrophages [144]. As well as this, MSC priming with another small molecule, the kinase inhibitor Ro-31-8425, has led to increased homing and anti-inflammatory effects [163].

## Considerations and perspectives

Despite great variability of MSC due to different culture protocols and tissue sources, the general immunosuppressive, immunomodulatory, and regenerative potentials of MSC are widely recognized (Fig. 1). However, several complications have limited the success of MSC therapy in clinical trials.

The high sensitivity of MSC to the harsh microenvironment of immune-mediated, inflammatory, and degenerative diseases is still a great obstacle for successful MSC-based therapies. Inhospitable tissue surroundings are able to limit the functions and survival of transplanted MSC. Thus, the use of “empowered” primed MSC may improve their therapeutic efficacy and expand their applications. Many other limitations have also jeopardized MSC-based therapies, such as cell senescence due to in vitro overexpansion, function loss after cryopreservation, and inconsistency of in vivo therapeutic effects among pre-clinical and clinical trials.

This scenario indicates how much new approaches are needed to improve MSC survival, proliferation and migration abilities, multilineage differentiation potential, immunosuppressive, immunomodulatory and regenerative functions, and therapeutic efficacy (Figs. 1 and 2).

In the last years, several priming approaches have been proposed to “empower” the therapeutic efficacy of MSC, with variable results. Noteworthy, MSC from distinct sources present variable responses to specific stimuli (priming factors or agents). In general, the majority of tested priming approaches were able to improve MSC proliferation, differentiation, and/or functions/therapeutic efficacy (Fig. 2).

However, priming approaches of MSC still have many limitations in the clinical translation, such as induction of immunogenicity, high costs, variable effects depending on MSC tissue source and donor variability, and lack of good manufacturing practices (GMP) grade certification for clinical application. Besides, the effect of priming approaches on the long-term tumorigenic potential of MSC has not been yet evaluated.

Further studies are currently needed to evaluate the (i) in vivo effects of different priming approaches; (ii) viability of cryopreserved primed-MSC; (iii) epigenetic modifications generated by specific priming approaches; (iv) efficacy of each priming strategy for different clinical applications; (v) best tissue sources for MSC isolation and best priming approaches for each clinical application; (vi) immunogenicity and tumorigenicity of primed and non-primed MSC; and (vii) proper universal potency assays for quality control of MSC products.

Researchers should also consider that MSC priming methods and agents must allow proper translation for clinical applications. The platform for the production of primed MSC should accomplish the criteria of quality cell therapy standards and allow cell expansion in clinical-grade scale (GMP), at the same time, not compromising the quality attributes of cells and not exceeding reasonable expenses.

## Abbreviations

5-aza-dC: 5-Aza-2′-deoxycytidine
ANG: Angiogenin
ANGPT-2: Angiopoietin-2
ANTXR1: Anthrax toxin receptor 1
APCDD1L: Adenomatosis polyposis coli downregulated 1-like
AR: Adenosine receptors
AS: Ankylosing spondylitis
AsAp: Ascorbic acid 2-phosphate
ATRA: All-trans retinoic acid
BAX: Bcl-2-associated X protein
BCL-2: B-cell lymphoma 2
BDNF: Brain-derived neurotrophic factor
BNIP3: BCL2-interacting protein 3
BST-2: Bone marrow stromal cell antigen 2
CCL: C-C motif chemokine ligand
CCPA: 2-Chloro-N6-cyclopentyl-adenosine
CDH1: Cadherin 1
Col-I: Type I collagen
CXCL: C-X-C motif chemokine ligand
CXCR: C-X-C chemokine receptor
DFO: Desferrioxamine
DHT: Dihydrotestosterone
DMOG: Dimethyloxalylglycine
DNP: 2,4-Dinitrophenol
ERK1/2: Extracellular signal-regulated kinase 1/2
FADS2: Fatty acid desaturase 2
FAK: Focal adhesion kinase
FGF: Fibroblast growth factor
FGL2: Fibrinogen-like protein 2
FN1: Fibronectin 1
G-CSF: Granulocyte-colony stimulating factor
GLUT: Glucose transporter
GRP78: 78-kDa glucose-regulated protein
H3K9: Histone H3 at lysine 9
HGF: Hepatocyte growth factor
HIF-1α: Hypoxia-induced factor-1 alpha
HK2: Hexo-kinase 2
HLA: Histocompatibility leucocyte antigen
HO-1: Heme oxygenase 1
ICAM-1: Intercellular adhesion molecule-1
IDO: Indoleamine 2,3-dioxygenase
IFN-γ: Interferon-gamma
IGF: Insulin growth factor
IKK: IκB kinase
IL: Interleukin
IL-17A: Interleukin-17A
IL-1α: Interleukin-1 alpha
IL-1β: Interleukin-1 beta
ISO: Isoflurane
ITGA: Integrin A
LDH: Lactate dehydrogenase
LDHA: Lactate dehydrogenase A
LPS: Lipopolysaccharide
MMP: Matrix metalloproteinase
MSC: Multipotent mesenchymal stromal cell
mTOR: Mammalian target of rapamycin
N-CAD: N-cadherin
NFκB: Factor nuclear kappa B
NKG2D: Natural killer group 2 member D
NOS2: Nitric oxide synthetase 2
NPR3: Natriuretic peptide receptor 3
Oct4: Octamer-binding transcription factor 4
PD-1: Programmed cell death protein-1
PDK1: Pyruvate dehydrogenase kinase 1
PDL-1: Programmed cell death-1 ligands
PEG: Poly(ethylene glycol)
PGA: Polyglycolic acid
PGE-2: Prostaglandin E2
PGF: Protenogenic growth factor
PKC: Protein kinase C
PLA: Polylactic acid
PLLA: Poly-l-lactic-acid
ROS: Reactive oxygen species
S1P: Sphingosine-1-phosphate
SDF-1: Stromal-derived factor 1
SEMA4D: Semaphorin-4D
SEMA7A: Semaphorin 7A
sHLA-G5: Soluble human leukocyte antigen-g5
Sox2: Sex-determining region Y-box 2
SOX9: Human SRY (sex determining region Y)-box 9
SP1: Sphingosine-1-phosphate
STAT1: Signal transducer and activator of transcription 1
STAT3: Signal transducer and activator of transcription 3
STC-1: Stanniocalcin-1
TGF-β1: Transforming growth factor β
TLR: Toll-like receptors
TNF-α: Tumor necrosis factor-alpha
TRAF-6: TNF receptor-associated factor 6
TSG-6: Tumor necrosis factor-inducible gene 6 protein
VCAM-1: Vascular cell adhesion molecule 1
VEC: Vascular endothelial cell
VEGF: Vascular endothelial growth factor
VPA: Valproic acid
