# Correction to: Priming approaches to improve the efficacy of mesenchymal stromal cell-based therapies

**DOI:** 10.1186/s13287-019-1259-0

**Published:** 2019-05-17

**Authors:** Nádia de Cássia Noronha, Amanda Mizukami, Carolina Caliári-Oliveira, Juçara Gastaldi Cominal, José Lucas M. Rocha, Dimas Tadeu Covas, Kamilla Swiech, Kelen C. R. Malmegrim

**Affiliations:** 1 Center for Cell-based Therapy, Regional Blood Center of Ribeirão Preto, Ribeirão Preto Medical School, University of São Paulo, Ribeirão Preto, Brazil; 2 Graduate Program on Bioscience and Biotechnology, School of Pharmaceutical Sciences of Ribeirão Preto, University of São Paulo, Ribeirão, Preto, Brazil; 3 In Situ Cell Therapy, SUPERA Innovation and Technology Park, Ribeirão Preto, São Paulo, Brazil; 4 Graduate Program on Basic and Applied, Immunology, Ribeirão Preto Medical School, University of São Paulo, Ribeirão, Preto, Brazil; 5 Department of Pharmaceutical Sciences, School ofPharmaceutical Sciences of Ribeirão Preto, University of São Paulo, Ribeirão, Preto, Brazil; 6 Department of Clinical, Toxicological and Bromatological Analysis, School of Pharmaceutical Sciences of Ribeirão Preto, University of São Paulo, Avenida do Café, s/n°, Ribeirão Preto, SP 14010-903 Brazil

**Correction to: Stem Cell Res Ther**

**https://doi.org/10.1186/s13287-019-1224-y**

The original article [1] contained an error in the presentation of the first author’s name, Nádia de Cássia Noronha. This has now been corrected.
